# FurinDB: A Database of 20-Residue Furin Cleavage Site Motifs, Substrates and Their Associated Drugs

**DOI:** 10.3390/ijms12021060

**Published:** 2011-02-08

**Authors:** Sun Tian, Qingsheng Huang, Ying Fang, Jianhua Wu

**Affiliations:** 1 Institute of Biomechanics, School of Bioscience and Bioengineering, South China University of Technology, Guangzhou 510006, China; E-Mails: huangqqss@163.com (Q.H.); yfang@scut.edu (Y.F.); 2 Nuolan Net, Wuhan 430071, China; 3 School of Life Sciences, Sun Yat-sen University, Guangzhou 510275, China

**Keywords:** furin, furin cleavage site, molecular database

## Abstract

FurinDB (freely available online at http://www.nuolan.net/substrates.html) is a database of furin substrates. This database includes experimentally verified furin cleavage sites, substrates, species, experimental methods, original publications of experiments and associated drugs targeting furin substrates. The current database release contains 126 furin cleavage sites from three species: mammals, bacteria and viruses. A main feature of this database is that all furin cleavage sites are recorded as a 20-residue motif, including one core region (eight amino acids, P6–P2′) and two flanking solvent accessible regions (eight amino acids, P7–P14, and four amino acids, P3′–P6′), that represent our current understanding of the molecular biology of furin cleavage. This database is important for understanding the molecular evolution and relationships between sequence motifs, 3D structures, cellular functions and physical properties required by furin for cleavage, and for elucidating the molecular mechanisms and the progression of furin cleavage associated human diseases, including pathogenic infections, neurological disorders, tumorigenesis, tumor invasion, angiogenesis, and metastasis. FurinDB database will be a solid addition to the publicly available infrastructure for scientists in the field of molecular biology.

## 1. Introduction

In the secretory pathway, many proteins are initially synthesized as inactive protein precursors, and proteolytic cleavage of these protein precursors by furin is an important step in the production of biologically active proteins [1,2]. Functionally, furin has two main subcellular localizations: the trans-Golgi network and the cell surface. In the trans-Golgi network, furin cleaves and activates most host substrates, including extracellular matrix proteins, signaling peptides, growth factors, serum proteins, cell surface transmembrane receptors and ion channels [1,2]. On the cell surface, furin cleaves many pathogenic substrates, such as bacterial toxins and viral fusion peptides. Maturation of functional proteins by furin cleavage is known to be involved in a wide range of human diseases, such as pathogenic infections, neurological disorders, tumorigenesis, tumor invasion, angiogenesis and tumor metastasis [1,2]. Recently, the use of the furin cleavage motif for targeted drug delivery in the treatment of tumors has been demonstrated [3].

**Figure 1.**
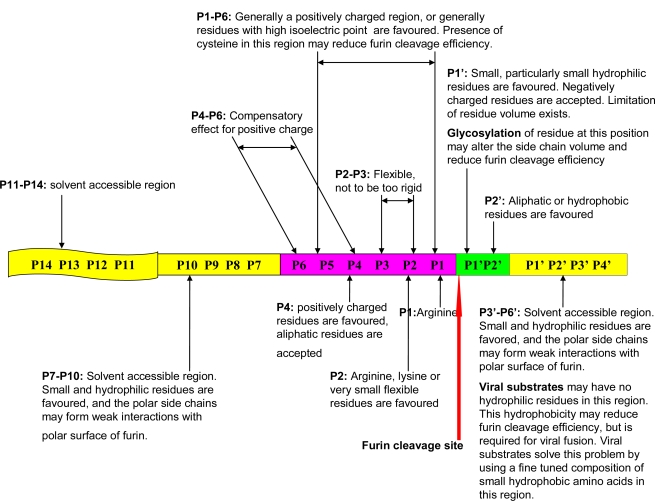
Physical properties of the 20 residue furin cleavage site motif. The physical properties of this motif have been conserved in molecular evolution. Distinct physical properties at different regions in the site affect the molecular biology of furin cleavage. The dynamic relationship between the physical properties of the P1′–P6′ region and viral infectivity is explained. The figure is reproduced from the author’s previous publication [4].

An important step in furin cleavage is the recognition of specific cleavage site motifs presented by furin substrates. Furin’s consensus cleavage site motif was widely reported to be a canonical four amino acid motif R-X-[KR]-R↓. In one of the author’s recent work, instead of this four amino acid motif, the furin cleavage site has been characterized as a 20 amino acid motif running from the P14–P6′ region, which can be divided into one core region (eight amino acids, P6–P2′) and two flanking solvent accessible regions (eight amino acids, P7–P14, and four amino acids, P3′–P6′) [4]. The backward numbering (P1–P14) and forward numbering (P1′–P6′) starts at the conserved arginine immediately prior to the furin cleavage site. The favored physical properties of each region required for interaction with the furin binding pocket and the solvent accessibility of furin substrates have been revealed (Figure 1) [4]. In mammals, bacteria and viruses, the evolutionarily conserved physical properties of furin cleavage site motifs are evident, and the dynamic relationship between the physical properties of the P1′–P6′ region and both cellular function and viral infection has been analyzed with respect to different species [4]. Using this updated understanding of the sequence motif and the molecular biology of furin cleavage, in this work, we have developed the FurinDB database. This database aims to facilitate further studies into the molecular biology of furin cleavage, the molecular mechanisms of its associated human diseases, and possible therapeutic treatments. The current version of FurinDB is a collection of 126 experimental data of known furin cleavage sites. It contains information about the amino acid residues in furin cleavage sites, the presence of multiple cleavage sites in the same protein, furin substrates, substrate species, experimental methods, original publications and associated drugs that target furin substrates. Substrates are grouped according to their biological functional categories and taxonomy.

We expect FurinDB to be a useful resource in the study of the molecular evolution and molecular biology of furin cleavage site motifs, as well as the elucidation of the molecular mechanisms and the progression of furin cleavage mediated human diseases, such as pathogenic infections, neurological disorders, tumorigenesis, tumor invasion, angiogenesis, and metastasis.

## 2. Results and Discussion

### 2.1. Database Content

Currently, the FurinDB database contains 126 cleavage sites from 110 substrates originating from three species [4], as well as 15 associated drugs that have either already been approved by the FDA or are in trials. All substrates, cleavage sites and original experimental records were collected from peer-reviewed literature [4]. The drug targets were identified by querying against Ingenuity Knowledge Base. The FurinDB database was developed using PHP 5 and MySQL database.

Each cleavage site is considered an entry, and each entry includes the following information:

1. Furin substrate sequence information: Name and description of the gene encoding the substrate protein and the substrate protein ID. External links to the NCBI protein sequence database and the NCBI Entrez gene database are also provided.
2. Furin cleavage site sequence information and location: The furin cleavage site P14–P6′ is represented as a 20 amino acids motif that contains one core region (eight amino acids, positions P6–P2′) packed inside the furin binding pocket; and two polar regions (eight amino acids, positions P7–P14; and four amino acids, positions P3′–P6′) located outside of the furin binding pocket [4]. The position P1, immediately prior to the furin cleavage site is recorded. The presence of multiple cleavage sites on the same protein substrate is indicated.
3. Taxonomy information of substrate: Each furin substrate is assigned to one of three taxonomy groups (mammals, bacteria or viruses).
4. Functional information about the furin substrates: Each furin substrate is assigned to one of seven predefined biological functions: (1) extracellular matrix protein; (2) signal peptide, hormone or growth factor; (3) serum protein; (4) transmembrane receptor; (5) viral protein; (6) bacterial protein or (7) other [1,2].
5. Experimental methods and conditions: Methods used to measure furin cleavage and the final cleavage products, the origin of the furin enzyme and the origin of the furin substrate used in the experiment and the literature record of the original experiments. These detailed experimental methods and conditions are grouped into 10 different types [5]. External links to the PUBMED literature database are also provided.
6. Associated drugs: Information is included for each furin substrate targeted by a drug that has either entered phase I trials or has already received FDA approval.

### 2.2. Database Access and Web Interface

The FurinDB database is publicly accessible through a web interface [5]. The website provides three different ways to facilitate searching the database, with different queries for gene name, biological function, taxonomy, drug availability and furin cleavage site motif.

Three different search methods have been implemented in the database:

1. Query by seven functional categories, three species and if the furin substrate is a known drug target.
2. Query by standard Entrez gene name.
3. Query by the eight amino acids motif in the core region P6–P2′ of the furin cleavage site, which is located inside the furin binding pocket. Either one or multiple amino acids can be input at any of the eight positions. The symbol X is interpreted to mean any amino acid.

**Figure 2.**
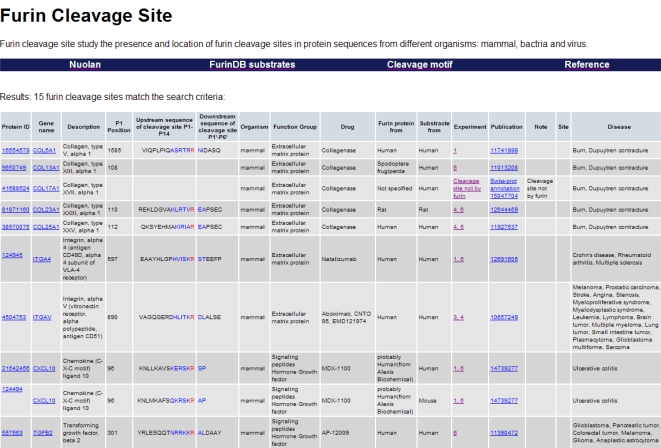
Screenshot of the FurinDB database. Information of substrates, cleavage sites, taxonomies, biological functions, experimental methods and associated drugs are displayed in table form.

Query results are displayed in table form (Figure 2). The core region (eight amino acids, positions P6–P2′), which is packed inside the furin binding pocket, is displayed in blue, and the arginine at position P1 immediately prior to the furin cleavage site is displayed in red.

## 3. Future Development

Future directions for development of the database include two parts: (1) We aim to collect additional furin substrates from the literature and extend this effort to include other members of the mammalian proprotein convertase family [6,7]. Furthermore, given the fact that many furin substrates are involved in growth, proliferation and extracellular matrix remodeling, we anticipate that more drugs, particularly anti-cancer drugs, will be developed to target furin substrates. This effort will be aided by the novel design of specific small molecular inhibitors targeting individual members of the mammalian proprotein convertase family. These specific small molecules are predicted to be derivatives of the short peptide pattern [K/R]-X-V-X-K-R [8]. Database information on furin substrate associated drugs will be regularly updated; (2) An algorithm for the prediction of location and presence of furin cleavage sites in protein sequences is currently being tested. The webserver for this prediction tool will be running along with the database, and it will also be freely available at the same web site which hosts the FurinDB.

## 4. Conclusions

We have developed FurinDB, a database of experimentally verified furin cleavage sites, substrates and associated drugs targeting furin substrates. All data is freely available at [5]. The database will be a valuable tool for studying the molecular biology and molecular evolution of furin cleavage sites, as well as elucidating the molecular mechanisms and progression of furin cleavage associated human diseases.
